# Advanced Placement Courses for Medical School: A Novel AMed Track to Reduce Financial Burden and Attract Nontraditional Students

**DOI:** 10.7759/cureus.18386

**Published:** 2021-09-29

**Authors:** Chase C Labiste, Kyle Huntley, Kyle A Bauckman, Lauren Fine, Vijay Rajput

**Affiliations:** 1 Internal Medicine, Nova Southeastern University Dr. Kiran C. Patel College of Allopathic Medicine, Fort Lauderdale, USA; 2 Orthopaedics, Nova Southeastern University Dr. Kiran C. Patel College of Allopathic Medicine, Fort Lauderdale, USA; 3 College of Allopathic Medicine, Nova Southeastern University Dr. Kiran C. Patel College of Allopathic Medicine, Fort Lauderdale, USA; 4 Medical Education, Nova Southeastern University Dr. Kiran C. Patel College of Allopathic Medicine, Fort Lauderdale, USA

**Keywords:** advanced placement, amed, three-year medical school, preclinical curriculum design and development, medical education

## Abstract

Medical school admissions have become increasingly competitive, creating a pool of nontraditional applicants that seek postbaccalaureate training in biomedical sciences. Several postbaccalaureate and graduate programs developed curricula that, except for learning clinical skills, mirror the learning objectives of the foundational science curricula in medical schools. This education structure provides applicants with a competitive advantage when applying to medical schools. However, basic science curriculum assessments in medical schools have changed to pass/fail scoring systems. As a result, students that participate in preparatory postbaccalaureate and graduate programs cannot show their superior level of knowledge and may find some core foundational science subjects redundant during their pre-clerkship medical education. The aim of this article is to propose an innovative system for matriculation into medical school through the AdvancedMed (AMed) Track, a three-year accelerated medical curriculum in which graduate curricula adopt an advanced placement course called AMed courses. This system would mirror the structure of the high school Advanced Placement (AP) system; therefore, students would take AMed courses similar in rigor to medical school basic science courses. These courses include Anatomy, Histology, Physiology, Cellular Biology, Biochemistry, Genetics, Microbiology, Immunology, Biostatistics, and Epidemiology. All courses would require a scored national standardized test to receive medical school credit toward a three-year accelerated track curriculum. Nontraditional students could choose to study independently and take the AMed standardized examination for credit to enter the AMed Track. Medical schools have the incentive to start an AMed Track because its implementation could lessen the financial burden, reduce time spent in medical school, and increase the participation of nontraditional medical students.

## Editorial

Introduction

Trends in medical education have led to the creation of shortened curricular pathways that reduce the duration of the medical school from the traditional four years to just three years. Since the inception of these three-year medical programs, there has been an expanding number of schools that offer such programs, each with their own motives and goals for doing so. Restrictive entry programs only allow select applicants that meet a specific criterion, such as previous doctoral training [1]. The restrictive exit models limit the types of residencies a student may apply to upon graduation. These programs benefit students by shortening their length of medical school. However, they require that the student choose a medical specialty prior to the beginning of clerkship education or upon matriculation. A benefit to three-year medical curriculum programs is to reduce the financial burden and overall debt [2]. The purpose of this article is to propose the AdvancedMed (AMed) Track, a new type of restrictive entry three-year medical curricula in which graduate curricula adopt an advanced placement course called AMed courses. These courses would have the same learning objectives of a foundational science section as a pre-clerkship medical school curriculum, except for clinical skills. After completing the AMed course or independently studying, students would take AMed standardized examinations, which would be similar to the Advanced Placement (AP) Examinations, because they could be used to assess a student’s knowledge about the subject.

AP programs began as a way to provide college-level learning to high school students, which reduced the credit requirements of incoming freshmen students. High school students take courses that have the same learning objectives as college-level courses. After completing an organized course or independently studying the material, the students take a scored national standardized examination that measures their competency in the area.

AMed Courses and Nontraditional Students

The AMed Track may also help increase participation from nontraditional medical school applicants. The definition of a nontraditional medical student varies across medical schools and in literature. For the purposes of this article, it will refer to students who are not accepted to medical school within a year of graduation with an undergraduate degree. The AMed Track could present an opportunity for those students who are worried about reduced earning potential and high student debt.

In recent years, the reduction of student debt has been a key focus of both students and university administrators. Medical school applicants, especially those that have completed postbaccalaureate programs and graduate degrees in biomedical science, also face a loss of value in the early years of pre-clerkship medical educations. Graduate and postbaccalaureate students who continue to medical school face additional costs compared to traditional medical students. They are responsible for the tuition of not only their graduate or postbaccalaureate program but also four years of traditional medical school. Therefore, these students who participate in postbaccalaureate and graduate programs are more likely to have increased premedical debt [3]. In addition, because many of these graduate and postbaccalaureate programs are designed to mimic the pre-clerkship curriculum rigor and subjects typical of medical school, these students could also find repetition and redundancy in the curricula. The AMed Track could have the dual benefit of reducing the amount of time spent in school and the amount of student debt as compared to students who complete graduate-level coursework prior to matriculation. The AMed Track could reduce the time spent learning basic science education so that those contact hours can be focused on clinical skills.

Pass/fail curricula, as opposed to score-based curricula, are now more common across medical education, especially during preclinical education. The shift toward a pass/fail curriculum during pre-clerkship years has de-emphasized the performance of medical students during their pre-clerkship curriculum. Moreover, the USMLE has announced that Step 1 will become pass/fail in 2022. Having a shortened pass/fail curriculum presents an opportunity for medical schools to shift toward a more clinically based style of learning for the pre-clerkship courses. The AMed Track could present an opportunity for schools to develop more clinically based style curricula. However, to succeed, this program would require that prospective students have a firm understanding of basic sciences prior to acceptance to medical school. Schools would benefit from using a standardized scoring system, like AP Examinations, that assesses a student’s likelihood to thrive in an accelerated program.

Proposed AMed Model

AMed Course Overview

The goal of an AMed course would be similar to that of a medical school course, in that students would be expected to know core medical concepts. These courses would be designed to achieve the same learning objectives expected by traditional medical schools. A standardized examination would be administered, similar to a high school system, in which students would be graded based on the requirements of medical education. Admission standards and requirements would be at the discretion of each medical school. Upon acceptance to medical school, the student would then be required to have met the score thresholds put in place by each school to satisfy the requirements to enter the three-year program.

Students that meet the score standards of the medical schools offering the AMed Track would undergo a year of medical education that emphasizes the subjects included in Table 1.

**Table 1:** Suggested AMed courses and three-year track core competencies.

AMed Courses	AMed Three-Year Track Courses
Anatomy/Histology	Pathophysiology of Diseases
Physiology/Cellular Biology	Pharmacotherapeutics
Biochemistry/Genetics	Clinical Skills
Microbiology/Immunology	Population Health
Biostatistics/Epidemiology	Ethics/Professionalism/Humanism/Leadership

These topics would be taught through a combination of problem-based learning, team-based learning, and clinical simulations through a systems-based approach. Pre-clerkship clinical learning sessions could be done in conjunction with the four-year cohort but may benefit from added AMed track-specific sessions to supplement learning objectives not covered during joint sessions. Students on this track would not receive additional lecture hours in the areas that they had received AMed credit. The intensive year prior to the beginning of clerkship education would emphasize collaboration among peers through clinical simulations. The students on this track would participate in problem-based and team-based learning sessions that would be separate from the standard curriculum. Therefore, students would still gain the benefits of learning to work collaboratively and practice communication skills. These students would have access to the same academic support resources as those students in the standard curriculum. Completion of this intensive year would distinguish students in this three-year program to begin clerkship education with other students who take the standard curriculum. Intentional thought about the integration of students on the AMed Track into the standard curricular activities should be given to reduce cost and enhance learning opportunities. 

AMed Courses and AMed Track for Graduate and Postbaccalaureate Students 

**Figure 1:**
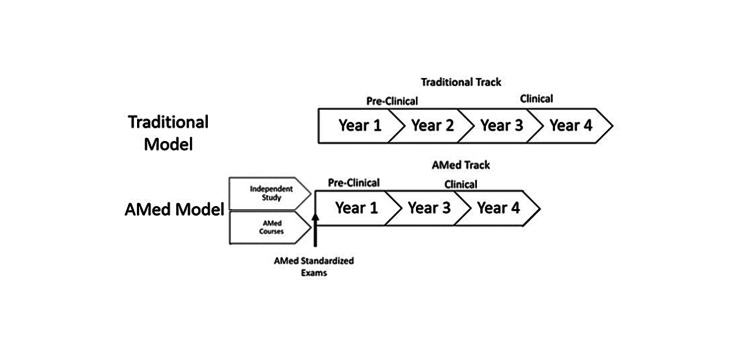
Standard medical school and AMed three-year medical school curriculum for postbaccalaureate, graduate students, and independent learners.

The goal of the AMed Track for graduate and postbaccalaureate programs would be to shift one year of medical education to the respective program. The implementation of AMed courses would most easily be through postbaccalaureate or biomedical master’s programs whose curricula are already designed to be equivalent to that of the first year of most medical schools. The students that enter these programs would seek to become more competitive for the medical school application process. The courses are designed to produce similar rigor to that seen in a typical medical school pre-clerkship curriculum. Postbaccalaureate and graduate programs would therefore be at an advantage to prepare their students to take the AMed standardized examination. The education standards for the AMed Track would require oversight from a governing medical education body such as the Association of American Medical Colleges (AAMC) or Liaison Committee on Medical Education (LCME) to produce detailed information on the topics that would be tested on the AMed standardized examinations. Figure 1 presents the proposed schematic.

AMed Standardized Examination

The AMed standardized examination would be available for aspiring medical students who are graduate, postbaccalaureate, or independent learners. The AMed standardized examinations would consist of multiple-choice questions designed by content experts in the fields relevant to the medical profession in the subjects in Table 1. They would be governed by the AAMC or another nationally recognized education or assessment board and administered in secure testing centers. 

AMed Track for Independent Learners

The AMed Track could also be available for independent learners. Students choosing to apply for this track could take the standardized examination after studying independently for the respective subjects. This track would be ideal for students with advanced degrees in scientific backgrounds. This system would mirror the ability of students to take AP standardized examinations without taking AP high school courses. These students would benefit from this type of program because they would not incur the cost of postbaccalaureate or graduate training. On the other hand, they would not benefit from participating in an academic program that mirrors the rigor of medical school. Figure 1 shows the proposed schematic for these types of students.

Discussion

The proposed AMed credit system would provide a solution to maintaining the quality of medical education while decreasing cost. This program would benefit medical education by reducing the financial burden, years spent in higher education, and increase the representation of nontraditional medical students. It would also provide students that excel in core medical competencies to reduce the time spent on a medical education while not sacrificing knowledge of basic sciences. In turn, medical schools could attract higher-achieving students.

The core AMed courses were chosen because they are essential to understand human structure and function. The redesigned curriculum would allow medical educators to focus on the pathophysiology and pharmacotherapeutics of disease. Students may also be able to contribute more in-depth knowledge during active small group learning. A drawback to implementing a shorter curriculum is the possibility that students have less time to learn essential knowledge needed for clinical skills, such as professionalism and communication. Although these students would have less time for the integration of core medical knowledge, they are expected to be on track with medical students in the standard program because the graduate programs have the same rigor and expectations. These students would benefit from being integrated into a clinical setting more quickly where they can spend time focusing on hands-on learning and improving their interpersonal skills.

The three-year program expects that there would be more well-qualified applicants than can be accepted. Students who are not accepted to the AMed Track could still have an advantage when applying to the standard medical school admission process. These students, if accepted through a traditional program, would be better equipped to face medical school-level courses than students who did not participate in AMed courses. The AMed courses could offer medical schools a better understanding of how that student would perform during the medical school curriculum. Standard medical school applicants that did not perform well during undergraduate science courses would have an opportunity to prove they can succeed in medical school-level courses. Therefore, students who choose to participate in AMed courses but do not get accepted to the AMed Track still have an advantage when applying to the standard medical education track. The AMed examinations could represent a standardized method to measure applicant competency in core medical subjects. 

The proposed AMed courses and AMed standardized examinations could present several limitations. First, this would require collaboration from multiple institutions that presents challenges such as time and money. The standardization of premedical post-undergraduate medical education would most likely benefit smaller, new programs. Well-established programs would be less likely to adopt changes to programs that have been successful for their students. However, the long-term benefit of implementing AMed courses would be the ability of graduate programs to advertise the success of their graduates using standardized scores in medical subjects.

A second potential limitation of the AMed model is the further exacerbation of disparities in attaining a medical education. Recent publications indicate that the benefit of high school AP courses may more directly assist students who are from White or higher-income families [4]. However, the population may still be at risk of being marginalized by an AMed independent study route. Postbaccalaureate training programs have also shown improved academic readiness and improve retention [5]. AMed could equalize the playing field and encourage minority medical school applications.

Conclusion

The primary aim of the AMed Track is to provide high-achieving students that excel in core medical competencies an avenue to reduce the time spent and financial burden associated with a medical education while not sacrificing knowledge of basic sciences. The AMed Track will succeed to the extent that there is national-level participation to establish education standards in conjunction with a governing body such as the AAMC or National Board of Medical Examiners. The positive outcomes of implementing AMed courses could extend beyond just to students who are accepted to the AMed Track. The program would create a standardized method to evaluate students that may have struggled academically during undergraduate courses. The AMed Track would also avoid the limitations that exit-restricted three-year accelerated programs have while also creating a much wider range of applicants than other entry-restricted programs that require higher education degrees. The proposed novel AMed courses and AMed three-year accelerated track are a possible solution to increasing the value of medical education for nontraditional medical students.
